# Identification of medicinal plants for the treatment of kidney and urinary stones

**DOI:** 10.15171/jrip.2016.27

**Published:** 2016-07-27

**Authors:** Mahmoud Bahmani, Babak Baharvand-Ahmadi, Pegah Tajeddini, Mahmoud Rafieian-Kopaei, Nasrollah Naghdi

**Affiliations:** ^1^Razi Herbal Medicines Research Center, Lorestan University of Medical Sciences, Khorramabad, Iran; ^2^Madani Heart Hospital, Department of Cardiovascular, Faculty of Medicine, Lorestan University of Medical Sciences, Khorramabad, Iran; ^3^Medical Plants Research Center, Shahrekord University of Medical sciences, Shahrekord, Iran; ^4^Clinical Microbiology Research Center, Ilam University of Medical Sciences, Ilam, Iran

**Keywords:** Kidney stones, Medicinal plants, Iran

## Abstract

**Introduction:** Kidney stones are the third most common urinary tract problems after urinary tract infections and prostate pathology. Kidney stones may cause extreme pain and blockage of urine flow. They are usually treated with medications that may cause a number of side-effects. Medicinal herbs are used in different cultures as a reliable source of natural remedies.

**Objectives:** This study aimed to determine native medicinal plants used by traditional healers of Shiraz for the treatment of kidney stones.

**Materials and Methods:** The ethno-medicinal data were collected between July and September 2012 through face-to-face interview with local herbalist.

**Results:** A total of 18 species belonging to 19 botanical families were recorded in study area. Species with the highest frequency of mentions were Alhagi maurorum (51.58%), Tribulus terrestris (51.58%), and Nigella sativa (48.14). The most frequently used plant parts were aerial parts (38%), leaf (33%) and fruits (17%). Decoction (68%) was the most frequently prescribed method of preparation. Most of the medicinal plants recommended by Shirazian herbalists have not been investigated in animal and humane models of renal stone which provides a new area of research.

**Conclusion:** In the case of safety and effectiveness, they can be refined and processed to produce natural drugs.

**Implication for health policy/practice/research/medical education:**

Kidney stones known as a renal calculus which it is a solid piece of material which is formed in the kidneys from minerals in urine. Kidney stones typically leave the body in the urine stream, and a small stone may pass without causing symptoms. The use of herbs in the prevention and treatment of kidney stones is a useful strategy. In this study, we have reported 18 species in Shiraz which were used for the treatment of renal calculus which could have the potential to produce natural remedies for removed kidney stone.

## Introduction

Kidney stones are the third most common urinary tract problems, after urinary tract infections and prostate diseases. Most people with kidney stones suffer from severe colic pains that are not relieved by conventional pain killers and may require narcotic analgesics. In addition to pain, urinary tract obstruction, urinary tract infection, hydronephrosis and severe bleeding may occur and in some cases, surgery is required to remove or break stones (1). The introduction of ESWL in the 1980s revolutionized the treatment of urinary stones. Today, more than 90% of patients with upper urinary tract stones are treated based on the size, type and location of the stone, with a treatment success rate of 68%-86% (2). It is has been reported that increased dietary protein intake may elevate the rates of developing kidney stones. Kidney stones are common clinical disorders and have both high incidence and high prevalence in the world. The prevalence of kidney stones is influenced by geographic location, lifestyle, race/ethnicity and other factors. In different studies, its world prevalence has been reported to be about 1%-15%. Iran has a high incidence of kidney stones prevalence. Approximately 75% of all kidney stones are calcium stone which composed of calcium oxalate and/or calcium phosphate (3).

It has been estimated that 80% of the world’s population relies on traditional medicine to treat their diseases (4). Medicinal plants have a long history of use and are globally safer than synthetic drugs (5). They are a reliable source for drug discovery (6). Today, researchers have focused on the drug discovery from medicinal plants (7). It has been estimated that at least one third of all medicinal product have plant origin (8). Medicinal plants are regarded as an acceptable, cheap, easily available and safe source of active compounds for pharmaceutical (9). The therapeutic effects of medicinal plants on kidney and urinary tract disorders have been variously studied and their efficacy has been demonstrated (10).

## Objectives

A wide variety of medicinal plants are used in Iranian traditional medicine to treat kidney disorders (11). This study aimed to determine the native medicinal plants used by traditional healers of Shiraz for the treatment of kidney stone.

## Materials and Methods

### The study area

This study was conducted in Shiraz which is located in the southwest of Iran. Shiraz is one of the largest cities in Iran and is the capital of Fars province. The city has a length of 40 km, a width of 15-30 km and a total area of 1268 km^2^. The population of this city was 1460665 in 2009. It has a moderate climate and lies in the Zagros mountain range at an altitude of 1468 m. It is surrounded by Kuh-e Sabz Pushan, Kuh-e Bamu, Kuh-e Chel Magham in the north and Kuh-e Drak in the west. The coldest month of the year is January, with an average temperature of 5℃ and the warmest month is July with an average temperature of 30℃. The average annual temperature is about 18℃ and the average annual rainfall is 3378 mm (12).

### The methodology of ethno-medicinal data collection

The ethno-medicinal data were collected between July and September 2012 through face-to-face interview with local herbalist and herbal healers. Herbalists were interviewed in their herbal stores with the aid of semi-structured questionnaires. Questionnaires were included herbalist personal information, plant local name, plant growth season, plant parts used, preparation methods, and traditional therapies. Questionnaires data were transferred to Microsoft Excel.

### Ethical issues

The research followed the tenets of the Declaration of Helsinki. The research was approved by the ethical committee of Shahrekord University of Medical Sciences.

### Statistical analysis

Data collected from local herbalist was analyzed using Microsoft Excel 2007.

## Results

**Figure 1:**
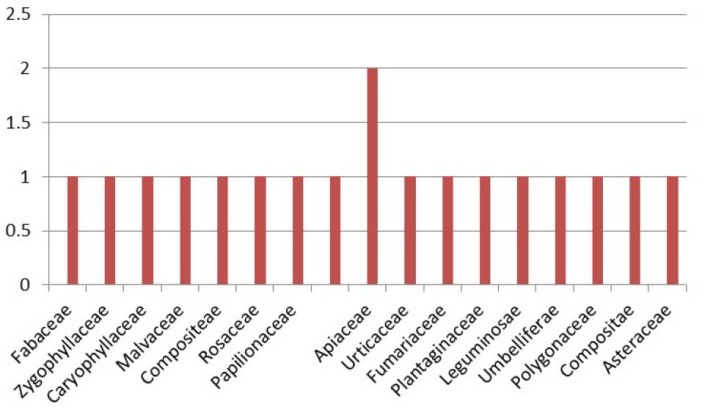
Botanical family recommended by Shirazian herbalist for the treatment of kidney stone.

**Figure 2:**
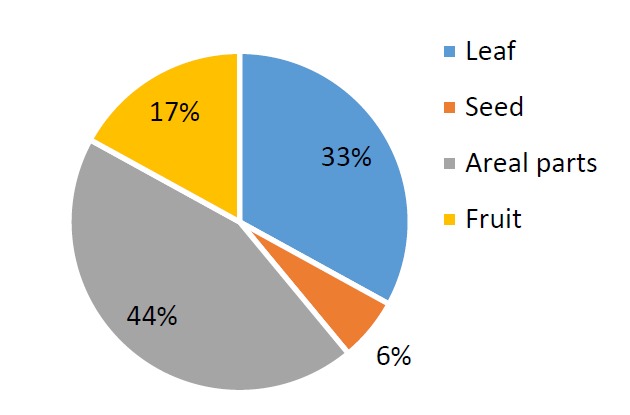
The percentage of different plant parts used to cure kidney stone in Shiraz.

**Figure 3:**
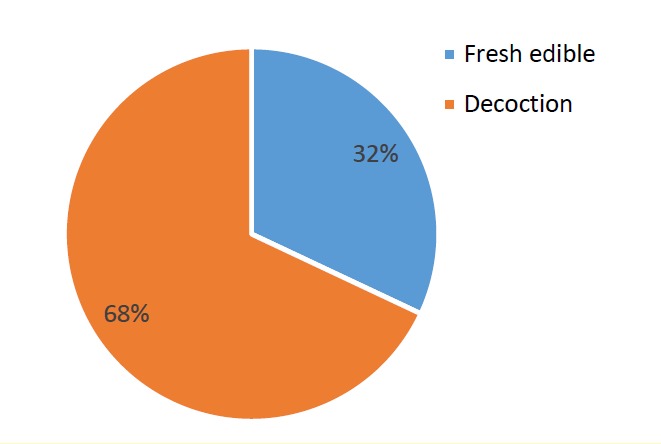
The percentage of different preparation methods of medicinal plant for the treatment of kidney stone in Shiraz.

Ethno-medicinal information of plants used in the management of kidney stone in Shiraz are shown in Table 1. A total of 18 species belonging to 19 botanical families are used to treat kidney stone in Shiraz. The number of mentions of each plant spices for the treatment of kidney stone is shown in Table 2. Species with the highest frequency of mentions in the interview were *Alhagi maurorum* (51.58%), *Tribulus terrestris* (51.58%), *Nigella Sativa* (48.14%), *Mangifera indica* (44.44%), *Prunus cerasus* (37.03%), *Prangos* acaulis (DC.) Bornm (33.33%). Botanical families recommended by Shirazian herbalist for the treatment of kidney stone are shown in Figure 1. Apiaceae was the most commonly recommended family. As shown in Figure 2 the most frequently used plant parts were aerial parts (38%), leaf (33%) and fruits (17%). Decoction (68%) was the most frequently prescribed method of preparation (Figure 3).

**Table 1:** Medicinal plant recommended for the treatment of kidney stone; scientific name, common name, family name, plant parts used and preparation methods

**Scientific name**	**Family**	**Persian names**	**Usable part of plant**	**How to use**	**Traditional therapeutic effect in Shiraz**
*Alhagi maurorum*	Fabaceae	Kharshotor	Aerial parts	Decoction	Kidney stone
*Tribulus terrestris*	Zygophyllaceae	Kharkhasak	Aerial parts	Decoction	Kidney stone
*Nigella Sativa*	Caryophyllaceae	Siahdaneh	Seed	Decoction	Kidney stone
*Althea aucheri Boiss.*	Malvaceae	Khatmi-armanestani	Aerial parts	Decoction	Kidney stone
*Lactuca sativa L*	Compositae	Kahoo	Leave	Fresh	Kidney stone
*Prunus cerasus*	Rosaceae	Albaloo	Fruit	Fresh	Kidney stone
*Alhagi camelorum*	Papilionaceae	Taranjebin	Aerial parts	Decoction	Kidney stone
*Mangifera indica*	Anacardiaceae	Anbeh	Fruit	Fresh	Kidney stone
*Prangos acaulis (DC.) Bornm*	Apiaceae	Jashi-kotoleh	Aerial parts	Decoction	Kidney stone
*Urtica dioica L*	Urticaceae	Gazaneh	Aerial parts	Decoction	Kidney stone
*Fumaria officinalis*	Fumariaceae	Shah-tareh	Leave	Decoction and fresh	Kidney stone
*Plantago psyllium*	Plantaginaceae	Esfarzeh	Leave	Decoction	Kidney stone
*Medicago sativa*	Leguminosae	Yonjeh	Decoction	Decoction	Kidney stone
*Apium graveolens*	Umbelliferae	Karafs	Decoction	Decoction	Kidney stone
*Rheum ribes*	Polygonaceae	Rivas	Fruit	Fresh	Kidney stone
*Arctium lappa*	Compositae	Baba-adam	Aerial parts	Decoction	Kidney stone
*Pimpinella anisum*	Apiaceae	Anison	Aerial parts	Decoction	Kidney stone
*Gundelia tournefortii*	Asteraceae	Kangar	Leave	Fresh	Kidney stone

**Table 2:** The number of mentions of each plant spices for the treatment of kidney stone

**Scientific name**	**The number of herbalists mentioned the plant**	**The total number of herbalists**	**Frequency of citation (FC) percentage (%)**
*Alhagi maurorum*	14	27	51.58
*Tribulus terrestris*	14	27	51.58
*Nigella Sativa*	13	27	48.14
*Althea aucheri Boiss.*	7	27	25.92
*Lactuca sativa L*	5	27	18.51
*Prunus cerasus*	10	27	37.03
*Alhagi camelorum*	12	27	44.44
*Mangifera indica*	9	27	33.33
*Prangos acaulis (DC.) Bornm*	2	27	7.40
*Urtica dioica L*	3	27	11.11
*Fumaria officinalis*	5	27	18.51
*Plantago psyllium*	4	27	14.81
*Medicago sativa*	5	27	18.51
*Apium graveolens*	4	27	14.81
*Rheum ribes*	2	27	7.40
*Arctium lappa*	4	27	14.81
*Pimpinella anisum*	2	27	7.40
*Gundelia tournefortii*	5	27	18.51

## Discussion

We collected local knowledge of Shirazian herbal healers on medicinal plants used in the treatment of kidney stone. A total of 18 species belonging to 19 botanical families are used to treat kidney stone in Shiraz. The most frequency used plant Species were *Alhagi maurorum* (51.58%), Tribulus terrestris (51.58%), *Nigella sativa* (48.14%), *Mangifera indica* (44.44%), *Prunus cerasus* (37.03%) and *Prangos acaulis (DC.).*

A wide variety of medicinal plants are used to treat renal stones in different parts of Iran. In ethno-botany of Kazeroon *Nasturtium officinale (L.) R. Br., Alhaji camelorum,* and *Tribulus terrestris L.* are used to treat kidney stone (13). *Alhagi persarum Boiss & Buhse and Rubia tinctorum* are used in Sistan and Baluchestan Province (southeastern Iran), to treat kidney stone (14). In Kashan ethnobotany, *Cousinia alexeenkoana Bornm* is used for this purpose (15). Kerman people believed that *Petroselinum hortense* can break up kidney stones (16). *Achillea santolina, Matricaria recutita L., Cuminum cyminum L., Nigella sativa L., Raphanus sativus L, Zea mays L., Plantago psyllium L., Linum usitatissimum L., Tribulus terrestris L., Prunus cerasus L and Foeniculum vulgare Mill* are used by Kurd tribe in Dehloran and Abdanan district, Ilam province for the treatment of kidney stones (17). *Adiantum capillus-veneris, Alhagi persarum Boiss, Allium akaka Gmelin, Cerasus mahaleb (L.) Miller, Gundelia tournefortii L.,* and *Noaea mucronata (Forssk.)* are traditionally used in Ilam Province as a means of breaking up kidney stones (18). Celery is widely used to treat kidney stone (19). Its essential oil contains chrysoeriol 7-O-diglucoside, Luteolin, 7-O-apiosylglucoside and Luteolin7-O (20).

A comparison of medicinal plants used in different parts of Iran shows that *Nigella sativa, Prunus cerasus, Tribulus terrestris*, and *Alhagi camelorum* are commonly used in different parts and cities for the treatment of kidney stones. The efficacy of some of these plants in treating kidney stone and other kidney diseases has been investigated in different studies (21,22). The effects of *Nigella sativa L* extract on ethylene glycol-induced kidney calculi in rats was investigated. Ethanolic extract of Nigella sativa significantly reduced the number of calcium oxalate deposits in rat kidney (23). In the study conducted by Shafaeifar et al, the effect of hydrophilic extract of *Alhagi maurorum* was investigated on ethylene glycol-induced renal stone formation in rats. Their results showed that the hydrophilic extract of *Alhagi maurorum* can reduce urinary oxalate concentration and urinary calcium oxalate stones formation (24). The effect of *Tribulus terrestris* extract on calcium oxalate crystallization in NRK 52E renal epithelial cells was investigated in the Aggarwal et al study. *Tribulus terrestris* extract significantly inhibited nucleation and the growth of the CaOx crystals (25).

Most of the medicinal plants recommended by Shirazian herbalist have not been investigated in animal and humane models of renal stone which provides a new area of research. In the case of safety and effectiveness, they can be refined and processed to produce natural drugs.

## Conclusion

In the case of safety and effectiveness, they can be refined and processed to produce natural drugs.

## Limitations of the study

This study limited to a Shiraz city. The same study in various parts of Iran suggests.

## Acknowledgments

The authors accomplish this research by the support of Shahrekord University of Medical Sciences, Shahrekord, Iran (Grant# 1842/5).

## Authors’ contribution

All the authors wrote the first draft of the manuscript equally. MRK revised and edited the last version.

## Conflicts of interest

The author(s) declared no potential conflicts of interest with respect to the research, authorship, and/or publication of this article**.**

## Ethical considerations

Ethical issues (including plagiarism, data fabrication, double publication) have been completely observed by authors.

## Funding/Support

The author(s) disclosed receipt of the following financial support for the research, authorship, and/or publication of this article: This article was prepared by support of Research Deputy of Shahrekord University of Medical Sciences.
